# Cytokine and Chemokine Profile in Patients with Multiple Myeloma Treated with Bortezomib

**DOI:** 10.1155/2020/1835836

**Published:** 2020-06-06

**Authors:** Paweł Robak, Edyta Węgłowska, Izabela Dróżdż, Damian Mikulski, Dariusz Jarych, Magdalena Ferlińska, Ewa Wawrzyniak, Małgorzata Misiewicz, Piotr Smolewski, Wojciech Fendler, Janusz Szemraj, Tadeusz Robak

**Affiliations:** ^1^Department of Experimental Hematology, Medical University of Lodz, Poland; ^2^Laboratory of Personalized Medicine, Bionanopark, Lodz, Poland; ^3^Department of Clinical Genetics, Medical University of Lodz, Poland; ^4^Department of Biostatistics and Translational Medicine, Medical University of Lodz, Poland; ^5^Department of Hematology, Medical University of Lodz, Poland; ^6^Department of Medical Biochemistry, Medical University of Lodz, Poland

## Abstract

The aim of the study was to determine the levels of selected cytokines and chemokines in the serum of multiple myeloma (MM) patients treated with bortezomib-based regimens. A total of 71 MM patients were examined: 41 with primary refractory disease (17) or early relapse (28), and 30 who were bortezomib sensitive with no progression for at least six months. Patients who demonstrated CR or PR after bortezomib-based therapies longer than six months after treatment discontinuation were designated bortezomib sensitive. Serum cytokine levels were assayed with Bio-Rad Bio-Plex Pro Human Cytokine 27-Plex Assay on the MAGPIX Multiplex Reader and the Bio-Plex® 200 System (Bio-Rad). Higher levels of MIP-1*α* and lower levels of MIP-1*β* and IL-9 were associated with better responses to bortezomib-based treatment, and higher levels of IL-1ra and IL-8 were associated with bone involvement. MCP-1 was elevated in patients with hemoglobin < 10 g/dl compared to those without anemia. The levels of IL-8, MIP-1*α*, and TNF-*α* were significantly higher in patients with renal insufficiency. Only MIP-1*α* was elevated in patients with hypercalcemia compared to patients with normal calcium levels. In conclusion, distinct cytokines are involved in the pathogenesis of MM and may play a prominent role in the prediction of treatment response. However, a single measurement of serum cytokines should be interpreted with caution and further studies are needed.

## 1. Introduction

Multiple myeloma (MM) is a plasma cell neoplasm with an annual incidence of 4.5-6 cases per 100,000 [1, 2]. In the United States, it is estimated that 32,110 new cases and 12,960 attributable deaths occurred in 2019. The disease is characterized by the malignant proliferation of monoclonal plasma cells in the bone marrow with a resultant elevation in monoclonal paraprotein and CRAB (calcium elevated, renal failure, anemia, and bone lesions) features [3]. Treatment of MM has changed dramatically in recent years, with the introduction of new drugs, especially proteasome inhibitors such as bortezomib, carfilzomib, and ixazomib [4]. Bortezomib is the first proteasome inhibitor that has become the standard of care in MM [5]. The drug exerts substantial antimyeloma activity in both previously untreated and relapsed/refractory MM patients, both when used as a single agent or in combination with other anticancer agents. However, most patients with MM who initially respond to bortezomib-based therapy eventually relapse and become resistant [6].

Cytokines play important roles in regulating immune responses. They are frequently produced by immune cells such as T cells, B cells, natural killer (NK) cells, neutrophils, and macrophages [7]. Three functional groups of cytokines can be distinguished, including T helper type 1 (Th1), Th2 cytokines produced by Th2 cells, and IL-17 family [8]. Th1 cytokines, produced by Th1 cells, play a role in cell-mediated immunity against intracellular bacterial and viral pathogens; Th2 cytokines, produced by Th2 cells, enhance humoral immunity against extracellular bacteria, parasites, toxins, and allergens; while Th17 cytokines defend the host from extracellular microorganisms such as fungi and bacteria [9]. Some cytokines, including interleukin- (IL-) 1, IL-6, IL-12, IL-15, IL-16, IL-17, IL-18, IL-22, IL-23, tumor necrosis factor-*α* (TNF-*α*), and interferon-*γ* (IFN-*γ*) have proinflammatory effects. In contrast, others like IL-1 receptor antagonist (IL-1ra), IL-4, IL-10, IL-11, and tumor growth factor *β*1 (TGF*β*1) exert anti-inflammatory activity [10]. Cytokines stimulate the proliferation, differentiation, and apoptosis of MM cells [11]. A network of cytokines is involved in the growth, progression, and dissemination of MM. They are also involved in MM-induced bone marrow destruction [10, 12, 13]. The bone marrow environment and MM cells stimulate paracrine or autocrine secretion of several cytokines; some of which can promote the growth, development, and progression of MM [14]. A recent study by Saltarella et al. found that the plasma levels of fibroblast growth factor-2 (FGF-2), hepatocyte growth factor (HGF), vascular endothelial growth factor (VEGF), and platelet-derived growth factor-*β* (PDGF-*β*) at diagnosis have predictive significance for response to treatment in MM-sensitive patients and those refractory to bortezomib [15]. The present study investigates the circulating levels of 27 cytokines, growth factors, and chemokines; their association with selected clinical and laboratory disease characteristics; and the response to bortezomib-based therapies.

## 2. Materials and Methods

The study group comprised 71 patients with MM treated at the Department of Hematology, Copernicus Memorial Hospital, Lodz, Poland from February 2016 to September 2019 (Table 1). The group comprised 43 men and 29 women with a mean age of 65 years (range, 39-91 years). The bone disease was evaluated using whole body computed tomography (CT) scan. All of the patients received bortezomib-based treatment as the first-line therapy. The patients included in the study were classified as bortezomib sensitive, bortezomib refractory, or early relapse according to their response to bortezomib-based therapy [16, 17]. The response to treatment and clinically meaningful evidence of progression were defined according to the International Myeloma Working Group (IMWG) [16, 18]. The response categories include complete response (CR), very good partial response (VGPR), partial response (PR), minimal response (MR), stable disease (SD), and progressive disease (PD). Disease progression was defined as a 25% increase in serum or urinary M-protein from the nadir levels documented at the time of best response [16]. A primary refractory patient was defined as a patient who received at least four courses of bortezomib-based therapies but did not demonstrate any CR, VGPR, or partial response (PR) or with documented progression during or within two months of completing treatment [17]. Patients who demonstrated at least PR within two months of completing treatment but with responses that lasted less than six months after the last dose of bortezomib were classified as early relapse. Patients who demonstrated CR, VGPR, or PR after bortezomib-based therapies later than six months after treatment discontinuation were designated bortezomib sensitive. In total, 13 patients were bortezomib refractory, 28 had early relapse, and 30 were bortezomib sensitive with no progression for at least six months of treatment discontinuation. The control group consisted of 30 healthy volunteers (12 women and 18 men; mean age 52.1 ± 8.8 years; range, 37-70 years).

The study was conducted according to good clinical and laboratory practice rules and the principles of the Declaration of Helsinki. All procedures were approved by the local ethical committee (The Ethical Committee of the Medical University of Lodz, No. RNN/103/16/KE). Each patient and control enrolled in the study gave written informed consent for all examinations and procedures.

### 2.1. Cytokine Analysis

Venous blood samples were collected between April 2016 and September 2018, and the patients were observed until September 2019. All samples were collected at diagnosis from treatment-naive patients. Patients with preexisting conditions (allergy/infections/therapy) that may cause changes in the cytokine profile were excluded before blood sample collection. After the collection of whole blood, it was left undisturbed at room temperature for 30 minutes. The samples were then centrifuged at 1000-2000 x g for 10 minutes in a refrigerated centrifuge. Serum samples from all studied patients and healthy individuals were stored at -70°C. The cytokine levels were examined at the end of the study, in order to avoid interassay variability.

Serum samples were tested using the Bio-Rad Bio-Plex Pro Human Cytokine 27-Plex Assay on the Bio-Rad MAGPIX Multiplex Reader for the following inflammatory cytokines: IL-1*β*, IL-1 receptor antagonist (IL-1ra), IL-2, IL-4 through IL-10, IL-12, IL-13, IL-15, IL-17, eotaxin (CCL11), fibroblast growth factor (FGF), granulocyte colony-stimulating factor (G-CSF), granulocyte macrophage colony-stimulating factor (GM-CSF), IFN-*γ*, IFN-*γ*-induced protein 10 (IP-10), monocyte chemoattractant protein 1 (MCP-1), macrophage inflammatory protein- (MIP-) 1*α* and MIP-1*β*, PDGF-BB, RANTES (Regulated on Activation, Normal T Expressed and Secreted, CCL5), TNF-*α*, and VEGF. The selection of evaluated cytokines and chemokines was based on previous studies in MM patients [10, 12, 19].

The assays are bead-based multiplex assays designed to measure these cell signaling proteins in diverse matrices. Based on the Luminex® xMAP® technology, the assays are capable of simultaneously quantifying 27 targets. Multiplex analysis gives the ability to look at analytes simultaneously providing more information from a lower sample volume in less time than traditional immunoassay methods. Similar to ELISA, xMAP utilizes an antibody sandwich for detection: however, it uses a different capture substrate and detection method. Bio-Plex® assays capture analytes in solution using differentially detectable bead sets and then employ fluorescent methods to identify them. Detection antibodies are then used to measure the quantity of analytes. The use of differentially detectable beads enables the simultaneous identification and quantification of many analytes in the same sample. The concentration of each cytokine was measured in relation to the calibration curve (individual for each cytokine) determined independently for each experiment (each plate). The samples were analysed in duplicate. In the last experiment, the analysis of a few samples (9) was repeated because the previously measured concentration of some cytokines was above the values included in the calibration curve. Then, 5-fold dilution of the serum samples was used instead of 4-fold.

### 2.2. Statistical Analysis

Nominal variables were expressed as percentages and analysed using the Chi-square test with appropriate corrections if needed: the Yates correction for continuity or Fisher's exact test. The normality of the distribution of continuous variables was verified with the Shapiro-Wilk test. Continuous variables were presented as medians with 25% to 75% values and compared using the Mann-Whitney *U*-test. The Kruskal-Wallis test was used for multiple comparisons, followed by the Mann-Whitney *U*-test with the Bonferroni correction for *post hoc* pair-wise comparisons. For the cytokines GM-CSF, IL-10, IL-12, and IL-15, the majority of measurements were below the limit of detection (LOD) and these values (75%, 51%, 63%, and 50%, respectively) were substituted by (1/2)∗LOD for statistical analyses. The same procedure was applied for IL-5 (28%), IFN-*γ* (20%), VEGF (14%), IL-6 (9%), IL-8 (5%), and IL-1*β*, IL-2, IL-7, IL-13, MIP-1*α*, and PDGF-BB, which were also below the level of detection (LOD). A similar approach was used in another cytokine-based study [20].

For a more comprehensive analysis, a logistic regression model was generated that used the response to a bortezomib-based regimen as outcome and the mediators as predictors. We estimated both a univariate model for each of the mediators and a multivariate model that included all selected predictors. A backward stepwise approach was used to restrict the model to the most predictive cytokines. The predictive power of the final model was evaluated by receiver operating characteristics (ROC) and area under the curve (AUC) analysis to determine the ability of the biomarker panel to accurately predict response to the bortezomib-based treatment regimen. Youden's index was applied to identify the optimal cut-off point. For the logistic regression, the mediator values were logarithmically transformed. The goodness of fit of the model was tested with the Hosmer-Lemeshow statistic, where a nonsignificant probability value indicates good fit. *p* values lower than 0.05 were considered statistically significant. All statistical analyses were conducted using Statistica Version 13.1 (TIBCO, Palo Alto, CA, USA).

## 3. Results

The demographic, clinical, and laboratory characteristics of the MM patients enrolled for the study are presented in Table 1. No statistically significant differences were observed in the following clinical findings: bone involvement at diagnosis (*p* = 0.84), calcium > 2.75 mmol/l at diagnosis (*p* = 0.13), creatinine > 2 mg/dl at diagnosis (*p* = 0.40), HB < 10 g/dl at diagnosis (*p* = 0.70), ISS (*p* = 0.99), age at initial bortezomib treatment (*p* = 0.11). The only statistically significant difference was observed in predominant paraprotein level (*p* = 0.031); in addition, light chain disease (LCD) was more common (36.7%) among the sensitive group than in the primary refractory group (7.7%) and the early relapse group (10.7%). It was found that 42 patients displayed IgG paraprotein, 14 demonstrated IgA, and 15 had LCD. No patients had received chemotherapy or radiotherapy prior to bortezomib treatment. Most of the patients (58–80.5%) received a bortezomib, cyclophosphamide, and dexamethasone (VCD) regimen, seven (9.7%) received VMP (bortezomib, melphalan, and prednisone), three (4.2%) received VTD (bortezomib, thalidomide, and dexamethasone), another three (4.2%) received VD (bortezomib and dexamethasone), and one received IsaVRd (isatuximab, lenalidomide, bortezomib, and dexamethasone). The levels of 27 cytokines were determined in all 71 MM patients treated with bortezomib-based regimens and the 30 controls. Thirty of the 71 patients were bortezomib sensitive, while the other 41 were primary refractory or early relapsed.

The cytokine profiles of the participants are summarized in Table 2 and Table S1 (Supplementary Materials). Compared to the controls, the MM patients demonstrated significantly higher serum levels of the following 14 cytokines: G-CSF, IL-1*β*, IL-4, IL-5, IL-6, IL-8, IL-9, IL-10, IL-15, IL-17A, IP-10, MIP-1*α*, MIP-1*β*, and RANTES (Table S1). In contrast, the levels of FGF basic, IFN-*γ*, IL-1ra, IL-7, PDGF-BB, and TNF-*α* were significantly decreased, and no significant difference was observed between the groups for eotaxin, GM-CSF, IL-2, IL-12, IL-13, VEGF, and MCP-1.

**Figure 1:**
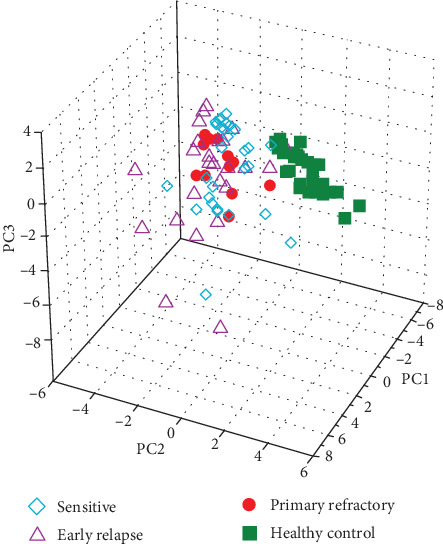
A principal component analysis (PCA) score in 3D plot of three multiple myeloma groups (sensitive, early relapsed, and primary refractory) and healthy controls.

The only significant difference observed between patients who were primary refractory to bortezomib and those with early relapse was that the level of eotaxin was higher in the former (91.35 pg/ml) than the latter (63.70 pg/ml, *p* = 0.006) (Table 2). No significant differences in any other cytokines or chemokines were observed between bortezomib-sensitive and bortezomib-refractory patients. Principal component analysis (PCA) was performed to increase the general understanding of the whole cytokine profile. The first three components cumulatively explain 52.5% of the variation in the data set, segregating the healthy control and all MM patients. Particular MM patient groups are not clearly separated (Figure 1).

**Figure 2:**
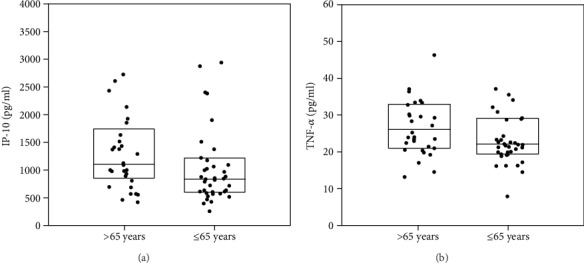
Significant differences of cytokine levels (IP-10 and TNF-*α*) in patients aged >65 and ≤65 years. The box plots depict the upper and lower quartiles and the median. (a) IP-10, *p* = 0.041; (b) TNF-*α*, *p* = 0.049.

**Figure 3:**
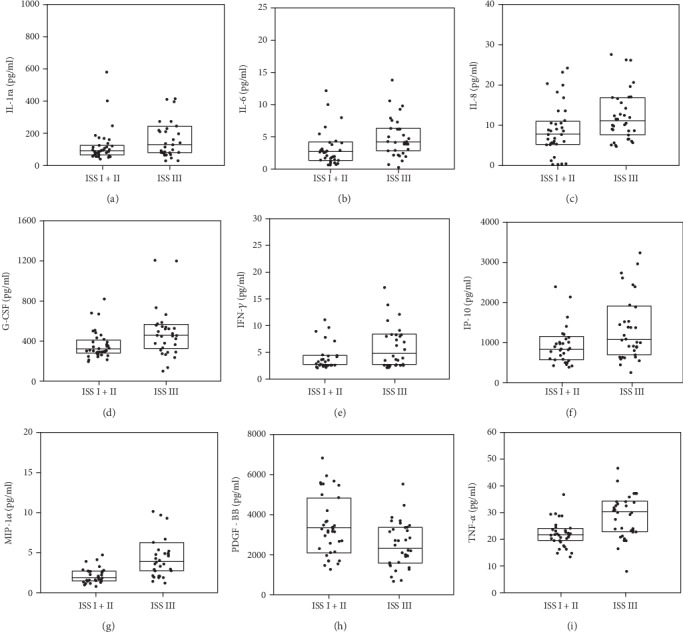
Significant differences of cytokine levels according to the International Staging System (ISS). The box plots depict the upper and lower quartiles and the median. (a) IL-1ra, *p* = 0.048; (b) IL-6, *p* = 0.008; (c) IL-8, *p* = 0.018; (d) G-CSF, *p* = 0.005; (e) IFN-*γ*, *p* = 0.019; (f) IP-10, *p* = 0.043; (g) MIP-1*α*, *p* < 0.001; (h) PDGF-BB, *p* = 0.008; (i) TNF-*α*, *p* = 0.001.

Comparisons of cytokine level according to gender, age, and International Staging System **(**ISS) are given in Table S2 (Supplementary Materials) and Figures 2 and 3. Generally, no significant difference was found between cytokine levels with regard to sex; however, the significantly higher levels of IP-10 and TNF-*α* were found in patients older than 65 years compared to younger patients (*p* = 0.041 and *p* = 0.049, respectively) (Figure 2). The levels of nine cytokines were significantly different between patients with more advanced ISS and those with less advanced ISS (Figure 3). The levels of IL-1ra, IL-6, IL-8, G-CSF, IFN-*γ*, IP-10, MIP-1*α*, and TNF-*α* were higher in patients with ISS III than in those with ISS I/II. Only PDGF-BB was lower in ISS III (2335.89 pg/ml) than in ISS I/II (3289.97 pg/ml, *p* = 0.049) (Table S2).

**Figure 4:**
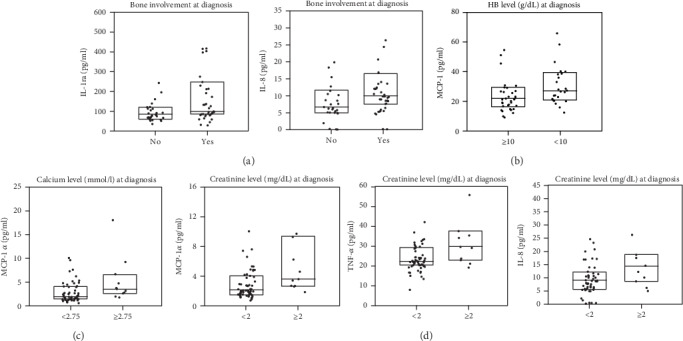
Significant differences of cytokine levels in patients according to CRAB symptoms. The box plots depict the upper and lower quartiles and the median. (a) Bone involvement at diagnosis: IL-1ra, *p* = 0.045; IL-8, *p* = 0.04. (b) HB level at diagnosis: MCP-1, *p* = 0.016. (c) Calcium level at diagnosis: MIP-1*α*, *p* = 0.023. (d) Creatinine level at diagnosis: MIP-1*α*, *p* = 0.013; TNF-*α*, *p* = 0.02; IL-8, *p* = 0.027.

Comparing the levels of cytokines with CRAB symptoms, i.e., hemoglobin, creatinine and calcium levels, and bone involvement (Figure 4 and Table S3, Supplementary Materials), it was found that patients with bone involvement displayed higher IL-1ra (98.94 pg/ml) than those without (84.44 pg/ml, *p* = 0.045) (Figure 4). The level of IL-8 was also higher in patients with bone involvement (10.01 pg/ml) than in those without (6.80 pg/ml; *p* = 0.040). In addition, 39 patients had bone involvement and 32 had no osteolytic changes at diagnosis. Anemia with HB < 10 g/dl was noted in 28 patients. Only MCP-1 level was higher in patients with anemia (28.21 pg/ml) than in those without (23.53 pg/ml; *p* = 0.016). Eleven patients had renal insufficiency, with ≥2 mg/dl creatinine; these patients demonstrated significantly higher levels of IL-8, MIP-1*α*, and TNF-*α* than those with <2 mg/dl creatinine (*p* = 0.027, *p* = 0.013, and *p* = 0.020, respectively) (Figure 4). Hypercalcemia, i.e., a calcium level ≥ 2.75 mmol/l, was observed in 11 patients; this group also demonstrated a significantly higher level of MIP-1*α* (3.57 pg/ml) than those with a normal calcium level (2.13 pg/ml; *p* = 0.023). Cytogenetics was performed in 38 patients (53.5% of all MM patients). Comparisons of cytokine level according to cytogenetic aberration demonstrated no statistically significant differences. The most common cytogenetic aberration in our study cohort was amp1q. Patients with this abnormality tend to have lower MCP-1 (*p* = 0.08) and higher IL-9 (0.096) and MIP-1*β* (0.099) levels.

**Figure 5:**
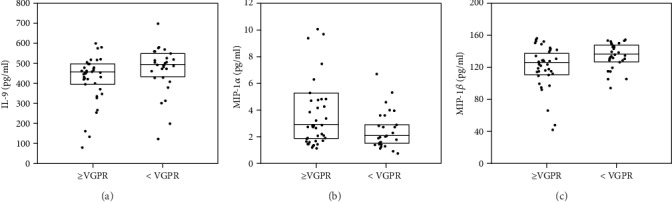
Cytokine levels according to response to treatment with bortezomib-based regimens (VGPR and higher or less than VGPR). Significant differences of three serum cytokine levels are shown. (a) IL-9, *p* = 0.045; (b) MIP-1*α*, *p* = 0.019; (c) MIP-1*β*, *p* = 0.022. The box plots depict the upper and lower quartiles and the median.

The levels of three cytokines (MIP-1*α*, MIP-1*β*, and IL-9) were dependent on the quality of responses to bortezomib-based treatment (Figure 5 and Table S4, Supplementary Materials). The level of MIP-1*α* was higher in patients who achieved CR (3.25 pg/ml) than a response less than CR (2.07 pg/ml, *p* = 0.037). MIP-1*β* levels were lower in patients with at least VGPR than less than VGPR (*p* = 0.022). The concentration of IL-9 was also lower in patients with at least VGPR (457.36 pg/ml) than those with less than VGPR (494.25 pg/ml, *p* = 0.045). Detailed information of all cytokine levels is shown in Table S4.

**Figure 6:**
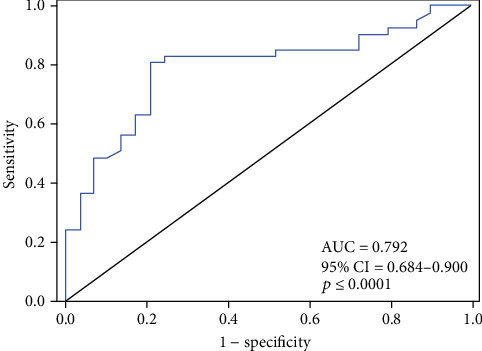
ROC curve analysis for the cytokine-based model in predicting response to bortezomib treatment in MM patients. At the optimal cut-off value of 0.53, the sensitivity and specificity reached 80.5% and 79.3%, respectively.

A logistic regression model was generated to evaluate the potential of serum cytokine profile for predicting response to a bortezomib-based treatment in MM patients. The final model is presented in Table 3. It consists of the three predictive cytokines chosen using the backward stepwise method. The Hosmer-Lemeshow test for goodness of fit indicated good calibration for the model (*p* = 0.087). A ROC analysis for the model (Figure 6) yielded an area under curve of 0.792 (95% CI: 0.684-0.900).

## 4. Discussion

Our findings indicate that MM patients possess significantly higher serum levels of G-CSF, IL-1*β*, IL-4, IL-5, IL-6, IL-8, IL-9, IL-10, IL-15, IL-17A, IP-10, MIP-1*α* and MIP-1*β*, and RANTES than those without MM. In addition, a number of cytokines including FGF basic, IFN-*γ*, IL-1ra, IL-7, PDGF-BB, and TNF-*α* were found to be significantly lower in the MM patients compared with healthy donors. Most of these observations are in agreement with previous studies [10, 12, 19, 21–26]. In particular, similar to our study, Sharma et al. report dysregulation in T helper 1/T helper 2 cytokine ratios in patients with MM: the serum levels of Th2 cytokines IL-4 and IL-10 were significantly elevated [26]. Interestingly, while the levels of the Th1 cytokines IFN-*γ* and IL-2 were found to be similar to those of normal controls in the present study, Sharma et al. found IFN-*γ* to be significantly reduced and IL-2 insignificantly increased.

In the present study, the IL-17A level in serum was found to be significantly higher in MM patients, and highest in those refractory to bortezomib. IL-17A has previously been found to be highly expressed in MM patients and to be able to induce MM cell viability [27], as well as to regulate osteoclast formation and activation [28]. It also promotes myeloma cell growth, inhibits immune function in MM through IL-17 receptor activity, and enhances adhesion to bone marrow stromal cells.

Our present findings indicate no significant difference in eotaxin level between MM patients and controls (*p* = 0.60); however, patients who were primary refractory to bortezomib demonstrated a higher eotaxin level than those in early relapse. Eotaxin is a member of the macrophage chemoattractant protein (MCP) subfamily; it acts as an immune modulator and a potent chemoattractant for cells, including eosinophils [29]. Its level has previously been found to be slightly elevated in MM/MGUS patients compared to controls, but not significantly so (*p* = 0.099) [30]. In addition, eotaxin level was significantly higher in the BM microenvironment of patients post allogenic stem cell transplantation (alloSCT) than in that of untreated MM patients [14]. These observations may suggest that eotaxin is not produced by myeloma cells but by nonmalignant inflammatory cells [31].

In the present study, the IP-10 serum level was found to be significantly higher in MM patients than in healthy controls (<0.001) (Table S1). IP-10 and its receptor CXCR3 have been shown to regulate the proliferation and survival of myeloma cells [32]. Bosseboeuf et al. did not observe any significant difference between MM patients and healthy controls (*p* = 0.114); however, their study included both patients with monoclonal gammopathy of undermined significance (MGUS) and those with MM in a single group [30]. Elsewhere, it was found that IP-10 is secreted by myeloma cell lines and was found to be increased in the BM environment of MM patients compared to healthy controls [14]. In addition, the concentrations of IP-10 in the BM correlated significantly with the stage of disease. These observations are consistent with our present findings, indicating that IP-10 serum level was significantly higher in stage III (1076.87 pg/ml) than in stage I/II (841.10 pg/ml, *p* = 0.043) (Table S2). In the present study, the levels of IL-1ra, IL-6, IL-8, G-CSF, IFN-*γ*, IP-10, MIP-1*α*, and TNF-*α* were also found to be higher in patients with ISS stage III than in those with ISS I/II. In previous studies, higher concentrations of IL-6, TNF-*α*, and IL-1*β* levels were also observed in the higher stages of the disease, suggesting an association with the proliferation of malignant plasma cells [23, 32].

CRAB factors distinguish active, symptomatic MM from MGUS and smoldering myeloma. These factors influence the prognosis of MM and are useful for deciding when to initiate treatment [18, 33]. In our group of patients, the most common CRAB factor was bone disease (55%), followed by anemia (40%), renal failure (15%), and hypercalcemia (15%). These frequencies are similar to those found in other studies [34]. Of these, bone disease has the strongest prognostic value, reflecting tumor burden and poor prognosis, even in the era of new drugs. In our study, only the levels of IL-1ra and IL-8 were higher in patients with bone involvement than in those without (Table S3). IL-8 increases osteoclast formation and contributes to bone metastasis [35, 36]. IL-1ra is a naturally occurring antagonist of IL-1*α*/IL-1*β* signaling pathways and has a modulating effect on the activity of osteoclast-activating factors [37]. However, the role of IL-1ra in the development of bone disease in MM patients requires further investigation.

In other studies, the level of IL-6 was found to be significantly elevated in MM patients who have at least three visible lytic bone lesions and/or bone fracture in comparison to patients with one or two visible or no visible lytic bone lesions (*p* = 0.048) [23]. IL-6 and IL-1*β* are potent osteoclast activators in myeloma pathogenesis. However, in our study both cytokines were found to have similar levels in patients with bone changes and those without. A previous study found that only one cytokine of a panel of 18 tested in bone marrow plasma from patients with MM, activin A, was significantly elevated among those with bone disease [38]. Other relevant cytokines, including IL-1*β*, IL-17, IL-32, and MIP-1*α*, were undetectable [39].

Anemia in MM can reflect both MM tumor burden and renal failure. Its incidence is believed to be related to the action of plasma cell-produced cytokines which inhibit erythropoiesis and impair iron homeostasis [40]. In the present study, MCP-1 was the only cytokine or chemokine whose level was higher in patients with anemia than in those without. Similar results have been reported by other authors. Valković et al. report a significant association between plasma MCP-1 level and more severe bone disease, renal impairment, and anemia [41]. MCP-1 is a potent chemoattractant for myeloma and other cells through its CCR2 receptor. It is expressed and released by a variety of cell types, including MM cells [42].

The levels of IL-8, MIP-1*α*, and TNF-*α* were significantly higher in patients with renal failure in comparison with patients with normal renal function (Table S3). In some studies, renal dysfunction was associated with a significant increase of serum cytokines, including IL-9 [19, 22]. IL-8 is a proinflammatory chemokine targeting neutrophils and lymphocytes that plays a significant role in inflammatory and tumor-associated angiogenesis and contributes to cancer progression through its induction of tumor cell proliferation, survival, and migration. In addition, IL-8 induces proliferation and chemotaxis among MM cells. In our study, serum IL-8 level was found to be higher in MM patients than in healthy controls, and higher in patients with kidney failure than in those with normal kidney function; similar findings have been reported previously [43].

Hypercalcemia in MM indicates a poor prognosis. However, in the current study, only the concentration of MIP-1*α* was higher in patients with high calcium level than in those with a normal level. Serum MIP-1*α* expression is known to correlate with survival and bone resorption markers, indicating that MIP-1*α* plays a role in the pathogenesis of bone disease in MM [44]. Previous studies have reported cases of hypercalcemia caused by high serum levels of MIP-1*α* and parathyroid hormone-related peptide (PTHrP) [45]. Increased MIP-1*α* serum level has also been described in mantle cell lymphoma and diffuse large cell lymphoma patients presenting with hypercalcemia and osteolysis [46, 47]. In MM patients, MIP-1*α* is a potent osteoclast-activating factor. It induces human osteoclast formation and is involved in bone destruction [48]. However, the molecular mechanism of MIP-1 involvement in hypercalcemia remains to be fully clarified.

The present study compares the levels of selected cytokines with the response to treatment with bortezomib-based regimens (Figure 3, Table S4). It was found that the level of MIP-1*α* was higher in patients who achieved CR than in those with less than CR. Similarly, MIP-1*α* was higher in patients with at least VGPR than in those with less than VGPR, and the concentrations of IL-9 and MIP-1*β* were lower in patients with at least VGPR than in those with less than VGPR. The relationship between the levels of serum cytokines and response to bortezomib-based therapies has not been reported in the literature so far. Higher serum PDGF-BB receptor levels have been observed in melphalan-resistant MM patients than in minor responding patients [49], and a recent study found that median serum platelet factor 4 (PF4) concentration was negatively associated with MM response and that serum PF4 level was significantly correlated with unfavorable clinical features [50]. Lower serum PF4 level was observed at diagnosis in MM patients who achieved CR and VGPR after two courses of VAD regimens, and IL-10 has been found to be a powerful predictor of prognosis for MM [51]. In patients treated with DVD (doxil, vincristine, and dexamethasone) or bortezomib-based regimens such as PAD (bortezomib, doxorubicin, and dexamethasone) and VTD (bortezomib, thalidomide, and dexamethasone), a higher serum IL-10 level (>169.96 g/ml) at diagnosis negatively correlated with progression-free survival (PFS) and overall survival (OS) [51]. Elsewhere, baseline VEGF serum levels were found to be significantly higher, and TNF-*α* serum levels significantly lower, in patients responding to treatment with thalidomide [48]. However, in the present study, the baseline serum levels of IL-10, VEGF, and TNF-*α* were similar in bortezomib-sensitive and bortezomib-refractory patients. A recent study found that the use of standard biomarkers, *viz.*, albumin, beta-2-microglobulin (*β*2M), paraprotein, and kappa/lambda (K/L) ratio, could facilitate a more personalized therapeutic approach and minimize unnecessary side effects from ineffective drugs [52]. It is possible that the effectiveness of this panel may be further enhanced by the addition of certain cytokines. Our present results were used to generate a multiple regression model that may have the potential to predict how patients with MM may respond to bortezomib-based therapy.

Our study has some limitations. It does not evaluate the levels of cytokines during or after therapy; however, other studies have found that plasma cytokines were not restored in patients in remission from MM [53]. A better understanding of the relationship between cytokines and response to treatment with modern therapies will ensure more effective therapeutic interventions in MM patients.

In conclusion, distinct cytokines are involved in the pathogenesis of MM. Better responses to bortezomib-based regimens were observed in patients with higher levels of MIP-1*α* and MIP-1*β*, and lower levels of IL-9. However, bortezomib sensitivity and refractoriness were not related to changes in cytokine levels. A single measurement of serum cytokines should be interpreted with caution and further studies are needed to elucidate their role in pathogenesis and prognosis of MM.

## Figures and Tables

**Table 1:** The characteristics of the patients treated with bortezomib-based therapy and healthy donors. Data are presented as frequency and percentage (%) unless otherwise specified.

Characteristics	Total	Primary refractory	Early relapse	Sensitive	Healthy donors
Number of patients	71	13	28	30	30
Gender *N* (%)	M: 42 (59.1) F: 29 (39.9)	M: 8 (61.5) F: 5 (38.5)	M: 17 (60.7) F: 11 (39.3)	M: 17 (56.7) F: 13 (43.3)	M: 18 (60.0) F: 12 (40.0)
Age at initial bortezomib treatment Mean + SD (range)	64.6 ± 11.2 (39-91)	69.1 ± 9.7 (46-81)	65.6 ± 12.0 (39-91)	61.6 ± 10.6 (42-82)	52.1 ± 8.8 (37-70)
Bortezomib regimen:					
VCD	58 (81.7)	10 (76.9)	21 (75.0)	27 (90.0)	—
VMP	6 (8.5)	2 (15.4)	4 (14.3)	0	—
VTD	3 (4.2)	0	1 (3.6)	2 (6.7)	—
VD	3 (4.2)	1 (7.7)	2 (7.1)	0	—
IsaVRd	1 (1.4)	0	0	1 (3.3)	—
Time from diagnosis to bortezomib treatment initiation—days—median (IQR)	16.5 (4-45)	96.0 (22-1356)	13.0 (4-39.5)	11.0 (3-35)	—
Paraprotein: *N* (%)					
IgG	42 (59.2)	10 (76.9)	16 (57.1)	16 (53.3)	—
IgA	14 (19.7)	2 (15.4)	9 (3.2)	3 (10.0)	—
LCD	15 (21.1)	1 (7.7)	3 (10.7)	11 (36.7)	—
Bone involvement at diagnosis	39 (54.9)	7 (53.8)	17 (60.7)	16 (53.3)	—
Calcium > 2.75 mmol/l at diagnosis	11 (15.5)	0	8 (28.6)	3 (10.0)	—
HB < 10 g/dl at diagnosis	28 (39.4)	6 (46.2)	8 (28.6)	14 (46.7)	—
Creatinine > 2 mg/dl at diagnosis	11 (15.5)	0	4 (14.3)	7 (23.3)	—
International Staging System (ISS) at diagnosis	I—18 (25.4)	I—4 (30.8)	I—7 (25.0)	I—7 (23.3)	—
	II—17 (23.9)	II—3 (23.1)	II—7 (25.0)	II—7 (23.3)	—
	III—34 (47.9)	III—6 (46.2)	III—13 (46.4)	III—15 (50.0)	—
CRP mg/l—median (IQR)	3.0 (1.0-7.4)	2.2 (1.1-5.9)	2.4 (1.3-6.3)	4.1 (1.0-7.3)	—
Beta-2-microglobuline increased (>3 mg/l)	49 (69.0)	9 (69.2)	21 (75.0)	19 (63.3)	—
LDH > 240 U/l	9 (12.7)	3 (23.1)	2 (7.1)	4 (13.3)	—
Cytogenetics—(%)	*N* = 38	*N* = 3	*N* = 18	*N* = 17	—
*t*(11; 14)	2.6	0	5.6	0	—
*t*(4; 14)	15.8	0	22.2	11.8	—
*t*(14; 16)	0	0	0	0	—
*t*(14; 20)	0	0	0	0	—
del(17p)	13.2	0	16.7	11.8	—
amp(1q)	55.3	33.3	61.1	52.9	—
del(13q)	23.7	0	16.7	35.3	—

Abbreviations: LCD—light chain disease; VCD—bortezomib, cyclophosphamide, and dexamethasone; VD—bortezomib and dexamethasone; VMP—bortezomib, melphalan, and prednisone; VTD—bortezomib, thalidomide, and dexamethasone; IsaVRd—isatuximab, lenalidomide, bortezomib, and dexamethasone; IQR—interquartile range.

**Table 2:** Cytokine profile of multiple myeloma (MM) patients and healthy volunteers. Data are presented as median values and 25% and 75% ranges, and as *p* values from global Kruskal-Wallis's test and *post hoc* comparisons (Mann-Whitney's tests, if the *p* value of the global test is significant). When *post hoc* tests were used with Bonferroni's correction, *p* values less than 0.0083 (0.05/6) were considered as significant. *p*^∗^:*p*values from MM patients who were profiled using Kruskal-Wallis's test only.

Cytokine (pg/ml)	N1 = 13 Primary refractory	N2 = 28 Early relapse	N3 = 30 Sensitive	N4 = 30 Healthy control	*p*	*p*∗	Cytokine level comparisons					
							N1 vs. N2	N1 vs. N3	N1 vs. N4	N2 vs. N3	N2 vs. N4	N3 vs. N4
FGF basic	36.76 (35.28; 40.98)	36.86 (30.05; 40.96)	37.39 (33.13; 44.36)	42.98 (38.19; 45.73)	0.002	0.422	0.327	0.947	0.010	0.240	**<0.001**	0.029
Eotaxin	91.35 (85.14; 122.48)	63.70 (41.35; 94.77)	80.60 (52.21; 114.19)	77.92 (64.39; 111.20)	0.020	0.016	**0.006**	0.128	0.059	0.094	0.036	0.912
G-CSF	370.56 (310.22; 452.35)	297.80 (404.66; 515.53)	285.18 (356.15; 565.95)	153.24; (125.02; 196.33)	<0.001	0.884	0.575	0.802	**<0.001**	0.883	**<0.001**	**<0.001**
GM-CSF	0.09 (0.09; 0.09)	0.09 (0.09; 1.07)	0.09 (0.09; 2.71)	0.09 (0.09; 0.09)	0.005	0.126	0.726	0.142	0.186	0.119	0.194	0.009
IFN-*γ*	2.56 (2.56; 3.92)	4.22 (2.58; 7.71)	3.17 (2.58; 8.03)	7.11 (5.48; 9.35)	0.001	0.528	0.300	0.634	**0.001**	0.460	0.022	**0.001**
IL-1*β*	1.18 (0.72; 1.40)	0.98 (0.79; 1.23)	0.96 (0.67; 1.58)	0.66 (0.56; 0.78)	<0.001	0.826	0.510	0.812	**0.002**	0.750	**<0.001**	**0.003**
IL-1ra	77.66 (56.52; 88.80)	123.61 (84.44; 229.09)	97.65 (81.76; 190.37)	156.76 (114.39; 200.03)	0.002	0.054	0.028	0.081	**0.001**	0.438	0.080	**0.004**
IL-2	5.29 (4.57; 6.59)	5.27 (4.45; 6.35)	5.80 (3.81; 8.09)	4.58 (3.75; 5.40)	0.088	0.591						
IL-4	6.10 (5.54; 8.14)	3.05 (4.32; 7.10)	5.89 (3.90; 8.04)	3.83 (3.46; 5.04)	0.001	0.042	0.022	0.321	**<0.001**	0.074	0.529	**0.004**
IL-5	21.91 (13.91; 24.73)	20.31 (13.93; 28.46)	18.70 (12.23; 29.20)	0.49 (0.49; 0.49)	<0.001	0.903	0.823	0.905	**<0.001**	0.663	**<0.001**	**<0.001**
IL-6	4.31 (3.71; 5.50)	2.77 (1.69; 4.53)	2.81 (1.32; 6.25)	0.78 (0.25; 1.15)	<0.001	0.256	0.096	0.182	**<0.001**	0.957	**<0.001**	**<0.001**
IL-7	24.31 (22.77; 30.93)	18.77 (12.76; 23.68)	21.56 (14.06; 27.80)	28.42 (23.61; 37.61)	<0.001	0.417	0.013	0.101	0.250	0.269	**<0.001**	**0.001**
IL-8	9.05 (6.45; 13.66)	10.04 (5.42; 14.00)	8.81 (5.61; 15.71)	5.47 (3.89; 7.84)	0.006	0.869	0.726	0.606	**0.004**	0.846	**0.006**	**0.008**
IL-9	521.17 (493.01; 563.54)	456.79 (388.65; 503.60)	463.19 (417.69; 498.60)	96.10 (77.64; 109.55)	<0.001	0.063	0.021	0.059	**<0.001**	0.669	**<0.001**	**<0.001**
IL-10	7.86 (0.70; 9.65)	0.70; (0.70; 10.18)	8.16 (0.70; 11.35)	0.70 (0.70; 2.73)	0.004	0.325	0.433	0.761	0.016	0.164	0.269	**0.001**
IL-12 (p70)	1.26 (0.52; 1.26)	0.52 (0.52; 1.85)	0.52 (0.52; 1.26)	0.52 (0.52; 0.52)	0.106	0.452						
IL-13	2.03 (1.21; 2.56)	1.95 (1.22; 2.37)	2.44 (1.59; 4.49)	2.25 (1.56; 2.93)	0.158	0.109						
IL-15	60.20 (0.73; 95.88)	33.09 (0.73; 57.22)	35.59 (0.73; 57.17)	0.73 (0.73; 17.63)	0.001	0.469	0.275	0.348	0.010	0.663	0.011	**0.001**
IL-17A	22.43 (18.08; 25.41)	17.54 (12.49; 22.80)	21.67 (14.22; 27.43)	15.31 (13.33; 17.86)	0.001	0.061	0.036	0.692	**<0.001**	0.061	0.118	**0.006**
IP-10	1005.09 (718.00; 1513.88)	995.24 (799.42; 1382.12)	856.17 (563.91; 1401.93)	446.74 (314.48; 532.69)	<0.001	0.246	0.751	0.552	**<0.001**	0.127	**<0.001**	**<0.001**
MIP-1*α*	1.98 (1.51; 2.27)	2.86 (1.86; 4.66)	2.61 (1.61; 4.85)	1.50 (1.04; 2.05)	<0.001	0.226	0.090	0.182	0.036	0.669	**<0.001**	**<0.001**
MIP-1*β*	144.24 (128.26; 150.91)	126.10 (109.80; 138.44)	126.92 (114.07; 138.88)	89.42 (73.77; 95.09)	<0.001	0.108	0.052	0.066	**<0.001**	0.715	**<0.001**	**<0.001**
PDGF-BB	1983.54 (1464.66; 2679.98)	2613.66 (1638.61; 3649.08)	3229.92 (2699.29; 3673.74)	3548.95 (2996.41; 4344.56)	0.003	0.065	0.293	0.016	**0.001**	0.222	**0.008**	0.145
RANTES	12785.10 (10177.75; 15066.21)	10531.33; (8930.91; 13155.06)	11470.88 (10461.28; 12495.46)	9020.47 (7730.21; 9708.91)	<0.001	0.123	0.052	0.321	**<0.001**	0.205	**0.001**	**<0.001**
TNF-*α*	23.98 (20.13; 29.36)	22.92 (20.94; 26.88)	23.41 (20.10; 36.45)	64.17 (49.52; 72.71)	<0.001	0.426	0.989	0.224	**<0.001**	0.319	**<0.001**	**<0.001**
VEGF	95.37 (73.59; 157.42)	78.35 (17.37; 147.09)	101.96 (77.96; 173.58)	112.53 (76.37; 181.90)	0.239	0.337						
MCP-1	26.35 (21.85; 29.66)	23.50 (16.46; 29.74)	23.65 (17.55; 36.64)	26.93 (16.21; 40.98)	0.733	0.624						

**Table 3:** Multivariate regression model for predicting response to bortezomib-based treatment in MM patients.

	Coefficient	OR	95% CI	*p* value
MIP-1*α*	1.870	6.490	2.192-19.217	0.001
IL-8	-0.768	0.464	0.228-0.943	0.034
IL-1*β*	-1.115	0.328	0.108-0.996	0.049

## Data Availability

All data are available from the corresponding author upon request.

## Supplementary Materials

Supplementary Materials Supplementary Table 1: cytokine profile of all multiple myeloma (MM) patients and healthy volunteers. Data are presented as median values and 25% and 75% ranges, and as *p* values from Mann-Whitney's test. Bonferroni's correction was used and *p* values less than 0.0083 (0.05/6) were considered as significant. Compared to the controls, the MM patients demonstrated significantly higher serum levels of 14 cytokines: G-CSF, IL-1*β*, IL-4, IL-5, IL-6, IL-8, IL-9, IL-10, IL-15, IL-17A, IP-10, MIP-1*α*, MIP-1*β*, and RANTES. The levels of FGF basic, IFN-*γ*, IL-1ra, IL-7, PDGF-BB, and TNF-*α* were significantly decreased, and no significant difference was observed between the groups for eotaxin, GM-CSF, IL-2, IL-12, IL-13, VEGF, and MCP-1. Supplementary Table 2: cytokine levels according to gender, age, and ISS. Data are presented as median and IQR. No significant difference was found between cytokine levels with regard to sex. Significantly higher levels of IP-10 and TNF-*α* were found in patients older than 65 years compared to younger patients (*p* = 0.041 and *p* = 0.049, respectively). The levels of IL-1ra, IL-6, IL-8, G-CSF, IFN-*γ*, IP-10, MIP-1*α*, and TNF-*α* were higher in patients with ISS III than in those with ISS I/II. Only PDGF-BB was lower in ISS III (2335.89 pg/ml) than ISS I/II (3289.97 pg/ml, *p* = 0.049). Supplementary Table 3: cytokine levels according to CRAB symptoms. Data are presented as median and IQR. Patients with bone involvement displayed higher IL-1ra (98.94 pg/ml) than those without (84.44 pg/ml, *p* = 0.045). The level of IL-8 was higher in patients with bone involvement (10.01 pg/ml) than in those without bone involvement (6.80 pg/ml; *p* = 0.040). MCP-1 level was higher in patients with anemia (28.21 pg/ml) than in those without (23.53 pg/ml; *p* = 0.016). Patients with renal insufficiency (creatinine ≥ 2 mg/dl) demonstrated significantly higher levels of IL-8, MIP-1*α*, and TNF-*α* than patients without renal insufficiency (creatinine < 2 mg/dl) (*p* = 0.027, *p* = 0.013, and *p* = 0.020, respectively). Hypercalcemia (calcium level ≥ 2.75 mmol/l) demonstrated a significantly higher level of MIP-1*α* (3.57 pg/ml) than those with a normal calcium level (2.13 pg/ml) (*p* = 0.023). Supplementary Table 4: cytokine levels according to response to treatment with bortezomib-based regimens. Data are presented as median and IQR. The level of MIP-1*α* was higher in patients who achieved CR (3.25 pg/ml) than in those who achieved a response less than CR (2.07 pg/ml, *p* = 0.037). MIP-1*β* levels were lower in patients with at least VGPR than in those with less than VGPR (*p* = 0.022). The concentration of IL-9 was also lower in patients with at least VGPR (457.36 pg/ml) than in those with less than VGPR (494.25 pg/ml, *p* = 0.045).
