# Supplementary material for: Cytokine and Chemokine Profile in Patients with Multiple Myeloma Treated with Bortezomib
Source: Mediators Inflamm. 2020 Jun 6;2020:1835836. doi: 10.1155/2020/1835836 (PMC7294367; doi:10.1155/2020/1835836)
Supplement: Supplementary Materials — Supplementary Table 1: cytokine profile of all multiple myeloma (MM) patients and healthy volunteers. Data are presented as median values and 25% and 75% ranges, and as p values from Mann-Whitney's test. Bonferroni's correction was used and p values less than 0.0083 (0.05/6) were considered as significant. Compared to the controls, the MM patients demonstrated significantly higher serum levels of 14 cytokines: G-CSF, IL-1β, IL-4, IL-5, IL-6, IL-8, IL-9, IL-10, IL-15, IL-17A, IP-10, MIP-1α, MIP-1β, and RANTES. The levels of FGF basic, IFN-γ, IL-1ra, IL-7, PDGF-BB, and TNF-α were significantly decreased, and no significant difference was observed between the groups for eotaxin, GM-CSF, IL-2, IL-12, IL-13, VEGF, and MCP-1. Supplementary Table 2: cytokine levels according to gender, age, and ISS. Data are presented as median and IQR. No significant difference was found between cytokine levels with regard to sex. Significantly higher levels of IP-10 and TNF-α were found in patients older than 65 years compared to younger patients (p = 0.041 and p = 0.049, respectively). The levels of IL-1ra, IL-6, IL-8, G-CSF, IFN-γ, IP-10, MIP-1α, and TNF-α were higher in patients with ISS III than in those with ISS I/II. Only PDGF-BB was lower in ISS III (2335.89 pg/ml) than ISS I/II (3289.97 pg/ml, p = 0.049). Supplementary Table 3: cytokine levels according to CRAB symptoms. Data are presented as median and IQR. Patients with bone involvement displayed higher IL-1ra (98.94 pg/ml) than those without (84.44 pg/ml, p = 0.045). The level of IL-8 was higher in patients with bone involvement (10.01 pg/ml) than in those without bone involvement (6.80 pg/ml; p = 0.040). MCP-1 level was higher in patients with anemia (28.21 pg/ml) than in those without (23.53 pg/ml; p = 0.016). Patients with renal insufficiency (creatinine ≥ 2 mg/dl) demonstrated significantly higher levels of IL-8, MIP-1α, and TNF-α than patients without renal insufficiency (creatinine < 2 mg/dl) (p = 0.027, p = 0.013, and p [file 1835836.f1.docx]

**Supplementary Materials**

­­­­­Cytokine and chemokine profile in patients with multiple myeloma treated with bortezomib

Paweł Robak^1^, Edyta Węgłowska^2^, Izabela Dróżdż^3^, Damian Mikulski^4^, Dariusz Jarych^2^, Magdalena Ferlińska^2^, Ewa Wawrzyniak^5^, Małgorzata Misiewicz^5^, Piotr Smolewski^1^, Wojciech Fendler^4^, Janusz Szemraj^6^, Tadeusz Robak^5^

**Table S1**. Cytokine profile of all multiple myeloma (MM) patients and healthy volunteers. Data are presented as median values and 25% and 75% ranges, and as p-values from Mann-Whitney test. Bonferroni’s correction was used and p-values less than 0.0083 (0.05/6) were considered as significant.

| **Cytokine** | **N1=71**  **All MM pants** | **N3=30**  **Healthy Control** | **p-value** |
| --- | --- | --- | --- |
| FGF Basic | 36,76  (35,28; 40,98) | 42,98  (38,19; 45,73) | **<0.001** |
| Eotaxin | 91,35  (85,14; 122,48) | 77,92  (64,39; 111,20) | 0.600 |
| G-CSF | 370,56  (310,22; 452,35) | 153,24;  (125,02; 196,33) | **<0.001** |
| GM-CSF | 1,79  (1,51; 2,20) | 1,09  (0,81; 1,36) | 0.028 |
| IFN-γ | 3,92  (3,61; 7,94) | 7,11  (5,48; 9,35) | **<0.001** |
| IL-1β | 1,18 (0,72; 1,39) | 0,66 (0,56; 0,78) | **<0.001** |
| IL-1ra | 77,66  (56,52; 88,80) | 156,76  (114,39; 200,03) | **0.002** |
| IL-2 | 4,57 (5,29; 6,59) | 4,58 (3,75; 5,40) | 0.017 |
| IL-4 | 6,10;  (5,54; 8,14) | 3,83 (3,46; 5,04) | **0.004** |
| IL-5 | 21,91 (13,91; 24,73) | 8,66 (4,10; 10,48) | **<0.001** |
| IL-6 | 4,31 (3,71; 5,50) | 1,00 (0,71; 1,29) | **<0.001** |
| IL-7 | 24,31  (22,77; 30,93) | 28,42  (23,61; 37,61) | **<0.001** |
| IL-8 | 9,05;  (6,45; 13,66) | 5,47 (3,89; 7,84) | **0.001** |
| IL-9 | 521,17 (493,01; 563,54) | 96,10 (77,64; 109,55) | **<0.001** |
| IL-10 | 9,57 (7,86; 13,43) | 3,17  (2,73; 4,49) | **0.003** |
| IL-12 (p70) | 1,26 (1,26; 1,96) | 1,95 (1,95; 3,46) | 0.079 |
| IL-13 | 2,03 (1,21; 2,56) | 2,25 (1,56; 2,93) | 0.580 |
| IL-15 | 94,55 (62,01; 126,99) | 25,37 (23,13; 32,69) | **<0.001** |
| IL-17A | 22,43 (18,08; 25,41) | 15,31 (13,33; 17,86) | **0.002** |
| IP-10 | 1005,09 (718,00; 1513,88) | 446,74 (314,48; 532,69) | **<0.001** |
| MIP-1α | 1,98 (1,51; 2,27) | 1,60 (1,05; 2,05) | **<0.001** |
| MIP-1β | 144,24 (128,26; 150,91) | 89,42 (73,77; 95,09) | **<0.001** |
| PDGF-BB | 1983,54 (1464,66; 2679,98) | 3548,95 (2996,41; 4344,56) | **0.003** |
| RANTES | 12785,10  (10177,75; 15066,21) | 9020,47 (7730,21; 9708,91) | **<0.001** |
| TNF-α | 23,98 (20,13; 29,36) | 64,17 (49,52; 72,71) | **<0.001** |
| VEGF | 119,75 (85,78; 212,95) | 115,69 (82,74; 181,90) | 0.146 |
| MCP-1 | 26,35 (21,85; 29,66) | 26,93 (16,21; 40,98) | 0.590 |

**Table S2**. Cytokine levels according to gender, age and ISS. Data are presented as median and IQR.

| **Cytokine**  [pg/mL] | **Gender** | | **p** | **Age** | | **p** | **ISS** | | **p** |
| --- | --- | --- | --- | --- | --- | --- | --- | --- | --- |
|  | **Male N=42** | **Female**  **N=29** |  | **>65 N=32** | **≤65**  **N=39** |  | **ISS III N=34** | **ISS I+II**  **N=37** |  |
| IL-1 β | 1.09  (0.75; 1.34) | 0.91  (0.70; 1.49) | 0.801 | 1.14  (0.72; 1.51) | 0.91  (0.62; 1.37) | 0.190 | 1.07 (0.72; 1.44) | 0.92  (0.75; 1.40) | 0.900 |
| IL-1ra | 96.17  (76.68; 167.15) | 95.06  (77.66; 190.37) | 0.972 | 122.88  (83.12; 221.44) | 94.71  (64.77; 156.91) | 0.113 | 130.49  (80.39; 247.91) | 88.80  (64.77; 123.61) | **0.048** |
| IL-2 | 5.38  (4.53; 7.59) | 5.46  (4.06; 6.59) | 0.930 | 5.76  (4.57; 8.33) | 5.23  (3.81; 6.59) | 0.135 | 5.93 (4.57; 8.58) | 5.23  (3.81; 6.59) | 0.216 |
| IL-4 | 5.92  (4.16; 7.70) | 5.42  (3.24; 7.92) | 0.245 | 5.82  (3.93; 8.33) | 5.81  (3.49; 7.42) | 0.329 | 5.84  (4.08; 7.92) | 5.85  (3.49; 7.42) | 0.475 |
| IL-5 | 19.41  (12.20; 27.96) | 19.86  (14.52; 28.95) | 0.587 | 23.05  (14.22; 29.08) | 19.09  (12.20; 25.46) | 0.253 | 24.05  (12.20; 29.87) | 17.95  (13.91; 24.73) | 0.311 |
| IL-6 | 2.81  (1.61; 4.96) | 4.01  (1.77; 6.25) | 0.406 | 3.49  (1.83; 6.33) | 2.73  (1.32; 4.31) | 0.335 | 4.21  (2.81; 6.33) | 2.60  (1.32; 4.16) | **0.008** |
| IL-7 | 22.76  (16.73; 27.82) | 19.55  (12.70; 24.31) | 0.149 | 22.77  (14.59; 29.93) | 21.99  (14.06; 25.85) | 0.446 | 22.77  (18.00; 26.05) | 21.99  (12.83; 27.80) | 0.384 |
| IL-8 | 9.49  (5.61; 15.71) | 8.72  (6.11; 12.48) | 0.757 | 10.33  (7.19; 16.79) | 8.72  (5.25; 11.58) | 0.092 | 11.09  (7.67; 16.83) | 7.77  (5.25; 11.14) | **0.018** |
| IL-9 | 465.98  (414.08; 518.82) | 465.23  (417.69; 520.13) | 0.757 | 478.02  (405.60; 519.81) | 464.15  (417.69; 518.82) | 0.632 | 440.96  (337.38; 519.49) | 479.63  (454.47; 518.82) | 0.087 |
| IL-10 | 1.29  (0.70; 10.87) | 8.46  (0.70; 10.27) | 0.292 | 9.30  (0.70; 12.06) | 0.70  (0.70; 8.84) | 0.066 | 8.22  (0.70; 10.72) | 6.66  (0.70; 11.35) | 0.657 |
| IL-12 (p70) | 0.52  (0.52; 1.26) | 0.52  (0.52; 1.26) | 0.669 | 1.26  (0.52; 2.38) | 0.52  (0.52; 1.26) | 0.202 | 0.52  (0.52; 1.26) | 0.52  (0.52; 1.61) | 0.787 |
| IL-13 | 2.05  (1.32; 2.61) | 2.03  (1.25; 3.33) | 0.752 | 2.00  (1.45; 2.68) | 2.15  (1.21; 2.92) | 0.972 | 2.13  (1.59; 2.70) | 2.01  (1.24; 2.92) | 0.820 |
| IL-15 | 35.59  (0.73; 61.65) | 38.46  (0.73; 60.20) | 0.870 | 42.27  (0.73; 61.04) | 31.60  (0.73; 63.14) | 0.377 | 42.18  (0.73; 63.82) | 31.60  (0.73; 57.17) | 0.270 |
| IL-17 | 20.14  (15.87; 24.50) | 18.31  (12.87; 24.86) | 0.377 | 22.35  (15.14; 26.79) | 18.31  (14.22; 23.39) | 0.257 | 22.54  (16.60; 28.13) | 18.84  (13.09; 23.94) | 0.142 |
| Eotaxin | 83.15  (55.89; 107.34) | 69.43  (41.92; 114.19) | 0.254 | 86.58  (50.62; 121.36) | 78.94  (49.35; 97.51) | 0.175 | 85.96  (58.96; 114.19) | 80.36  (49.35; 100.63) | 0.468 |
| FGF basic | 38.55  (32.11; 42.89) | 35.28  (32.11; 40.01) | 0.211 | 39.59  (34.20; 43.36) | 36.76  (30.82; 40.61) | 0.061 | 38.33  (58.96; 114.19) | 37.02  (30.82; 40.98) | 0.355 |
| G-CSF | 367.49  (297.12; 521.64) | 374.96  (292.18; 479.70) | 0.458 | 404.66  (308.78; 534.74) | 338.55  (285.18; 503.21) | 0.265 | 457.88  (328.32; 565.95) | 326.01  (281.59; 413.91) | **0.005** |
| GM-CSF | 0.09  (0.09; 2.09) | 0.09  (0.09; 2.06) | 0.563 | 0.09  (0.09; 2.05) | 0.09  (0.09; 2.50) | 0.682 | 0.09  (0.9; 2.20) | 0.09  (0.09; 2.09) | 0.848 |
| IFN- γ | 2.92  (2.58; 7.10) | 3.61  (2.58; 8.03) | 0.516 | 3.76  (2.58; 8.13) | 3.16  (2.58; 5.52) | 0.755 | 4.91  (2.58; 8.36) | 2.67  (2.58; 4.29) | **0.019** |
| IP-10 | 877.46  (631.97; 1370.52) | 1093.28  (696.74; 1895.99) | 0.139 | 1109.96  (845.16; 1748.96) | 841.10  (597.72; 1216.65) | **0.041** | 1076.87  (688.24; 1895.99) | 841.10  (564.77; 1123.93) | **0.043** |
| MCP-1 | 22.71  (18.05; 36.29) | 27.46  (20.01; 33.14) | 0.465 | 24.47  (18.17; 38.69) | 23.63  (18.05; 33.14) | 0.623 | 26.09  (18.78; 36.64) | 21.85  (17.38; 33.14) | 0.352 |
| MIP-1 α | 2.67  (1.86; 4.15) | 2.60  (1.58; 4.74) | 0.708 | 2.81  (1.79; 4.83) | 2.62  (1.61; 4.15) | 0.402 | 3.91  (2.68; 6.27) | 1.86  (1.43; 2.73) | **<0.001** |
| PDGF- BB | 3018.94  (1928.80; 3502.82) | 2699.29  (1907.10; 3673.74) | 0.902 | 2515.66  (1775.99; 3501.92) | 3145.72  (1953.27; 3645.86) | 0.335 | 2335.89  (1566.97; 3350.41) | 3289.97  (2094.77; 4832.14) | **0.008** |
| MIP-1 β | 127.07  (114.07; 142.83) | 127.94  (114.49; 143.08) | 0.801 | 130.98  (114.28; 143.66) | 126.86  (112.85; 140.95) | 0.552 | 126.78  (105.35; 138.88) | 127.94  (115.36; 144.69) | 0.267 |
| RANTES | 11470.88  (10164.42; 13834.85) | 10918.98  (9811.60; 12785.10) | 0.563 | 11034.46  (9880.46; 14100.28) | 11425.05  (9915.60; 12785.10) | 0.786 | 11020.79  (9319.77; 12780.70) | 11760.91  (10164.42; 13834.85) | 0.359 |
| TNF- α | 23.40  (20.10; 32.32) | 22.93  (20.85; 29.57) | 0.916 | 26.11  (21.13; 32.80) | 22.00  (19.46; 28.94) | **0.049** | 29.86  (22.63; 33.95) | 21.67  (19.46; 23.98) | **0.001** |
| VEGF | 87.19  (51.73; 149.14) | 101.29  (61.59; 173.58) | 0.490 | 87.73  (53.70; 142.58) | 103.08  (57.45; 212.95) | 0.306 | 102.18  (73.59; 157.42) | 85.78  (2.40; 173.58) | 0.277 |

**Table S3.** Cytokine levels according to CRAB symptoms. Data are presented as median and IQR.

| **Cytokine**  [pg/mL] | **Bone involvement at diagnosis** | | **p** | **HB level (g/dL) at diagnosis** | | **p** | **Creatinine level (mg/dL) at diagnosis** | | **p** | **Calcium level at diagnosis (mmol/l)** | | **p** |
| --- | --- | --- | --- | --- | --- | --- | --- | --- | --- | --- | --- | --- |
|  | **Yes**  **N=39** | **No**  **N=32** |  | **<10**  **N=28** | **≥10  N=43** |  | **≥2**  **N=11** | **<2**  **N=60** |  | **≥2.75 N=11** | **<2.75 N=60** |  |
| IL-1 β | 1.03  (0.72; 1.37) | 0.92  (0.70; 1.49) | 0.960 | 1.06  (0.71; 1.29) | 0.92  (0.60; 1.49) | 0.884 | 1.02  (0.84; 1.54) | 0.92  (0.70; 1.38) | 0.436 | 1.03  (0.71; 1.23) | 0.92  (0.70; 1.42) | 0.666 |
| IL-1ra | 98.94  (81.91;247.91) | 84.44  (64.77; 122.16) | **0.045** | 92.39  (73.18; 203.91) | 94.71  (71.30;127.55) | 0.634 | 211.82  (64.82; 275.30) | 93.34  (73.99; 135.17) | 0.181 | 229.09 (64.82; 413.49) | 93.34  (73.99; 135.17) | 0.126 |
| IL-2 | 5.46  (4.57; 6.60) | 5.08  (3.81; 7.76) | 0.705 | 5.26  (4.55; 7.16) | 5.08  (4.05; 6.59) | 0.666 | 4.99  (3.03; 8.58) | 5.26  (4.21; 6.59) | 0.859 | 5.93  (4.05; 6.62) | 5.15  (4.21; 7.15) | 0.892 |
| IL-4 | 5.81  (3.58; 7.97) | 5.78  (3.70; 7.06) | 0.946 | 5.69  (3.64; 7.98) | 5.81  (3.41; 7.06) | 0.814 | 4.61  ( 3.06; 7.79) | 5.83  (3.58; 7.24) | 0.431 | 4.61  (3.06; 8.52) | 5.76  (3.64; 6.92) | 0.946 |
| IL-5 | 19.09  (13.34; 29.93) | 19.73  (13.91; 25.46) | 0.696 | 19.41 (13.63; 29.53) | 19.09  (12.23; 25.85) | 0.680 | 24.73  (13.34; 30.31) | 18.70  (13.01; 25.85) | 0.322 | 27.96  (12.21; 29.93) | 18.70  (13.45; 25.37) | 0.210 |
| IL-6 | 3.83  (1.92; 6.20) | 2.92  (1.32; 4.30) | 0.295 | 3.37  (1.97; 6.88) | 2.96  (1.48; 4.31) | 0.438 | 3.86  (2.92; 4.75) | 2.77  (1.45; 5.10) | 0.388 | 3.86  (2.52; 7.56) | 2.81  (1.45; 4.95) | 0.326 |
| IL-7 | 22.77  (15.42; 27.80) | 22.34  (14.06; 25.85) | 0.705 | 21.75  (14.63; 28.19) | 22.34  (14.06; 25.85) | 0.634 | 20.78  (18.03; 24.31) | 22.53  (13.44; 26.70) | 0.735 | 22.77  (14.06; 24.31) | 22.16  (14.06; 26.60) | 0.953 |
| IL-8 | 10.01  (7.67; 16.75) | 6.80  (5.25; 11.58) | **0.040** | 8.89  (6.54; 12.39) | 8.72  (5.25; 14.26) | 0.434 | 14.26  (8.56; 18.47) | 8.64  (5.59; 11.76) | **0.027** | 10.60  (8.56; 14.26) | 8.64  (5.59; 13.23) | 0.173 |
| IL-9 | 459.02 (372.13; 518.82) | 479.63 (432.01; 529.40) | 0.181 | 464.23  (427.14; 535.59) | 476.02  (414.08; 518.82) | 0.834 | 417.69  (315.19; 498.60) | 476.47  (433.59; 520.65) | 0.061 | 422.28 (337.38; 479.12) | 478.28 (433.58; 520.65) | 0.069 |
| IL-10 | 7.86  (0.70; 10.71) | 0.70  (0.70; 10.27) | 0.531 | 7.29  (0.70; 9.66) | 0.70  (0.70; 11.78) | 0.819 | 1.87  (0.70; 9.66) | 7.08  (0.70; 9.96) | 0.946 | 0.70  (0.70; 11.78) | 6.88  (0.70; 9.65) | 0.946 |
| IL-12 (p70) | 0.52  (0.52; 1.26) | 1.26  (0.52; 1.96) | 0.457 | 1.26  (0.52; 2.20) | 0.52  (0.52; 1.26) | 0.105 | 1.26  (0.52; 2.65) | 0.52  (0.52; 1.26) | 0.173 | 0.52  (0.52; 1.26) | 0.52  (0.52; 2.13) | 0.352 |
| IL-13 | 2.03  (1.25; 2.67) | 2.09  (1.24; 3.44) | 0.799 | 2.09  (1.45; 2.70) | 1.97  (1.21; 2.67) | 0.666 | 2.61  (1.66; 3.25) | 1.98  (1.22; 2.61) | 0.148 | 2.21  (1.13; 2.67) | 2.00  (1.24; 2.70) | 0.872 |
| IL-15 | 34.57  (0.73; 60.20) | 42.18  (0.73; 63.82) | 0.931 | 36.52  (0.73; 61.04) | 38.43  (0.73; 63.82) | 0.919 | 34.57  ( 0.73; 63.82) | 38.45  (0.73; 61.04) | 0.852 | 34.57  (0.73; 44.94) | 38.45  (0.73; 63.48) | 0.678 |
| IL-17 | 20.27  (14.22; 23.94) | 17.99  (15.12; 24.86) | 0.931 | 18.20  (14.98; 25.60) | 18.88  (13.09; 24.23) | 0.731 | 18.30  (14.22; 26.15) | 18.86  (14.56; 24.22) | 0.839 | 18.30  (14.22; 23.13) | 18.86  (14.56; 24.68) | 0.966 |
| Eotaxin | 80.94  (49.66; 110.45) | 80.36  (49.35; 107.34) | 0.901 | 79.89  (49.50; 108.90) | 80.36  (49.06; 103.43) | 0.854 | 69.43  (49.06; 114.19) | 80.60  (49.21; 102.03) | 0.819 | 66.32  (49.06; 117.97) | 80.42  (49.21; 105.38) | 0.926 |
| FGF basic | 38.29  (33.13; 41.72) | 35.85  (32.11; 40.98) | 0.472 | 37.36  (32.21; 43.69) | 36.76  (29.49; 40.61) | 0.283 | 35.85  (33.13; 40.94) | 37.05  (31.47; 41.05) | 0.899 | 40.61  (32.11; 42.89) | 36.89  (31.84; 40.94) | 0.600 |
| G-CSF | 381.98 (296.79; 503.21) | 347.87 (277.67; 504.90) | 0.620 | 383.83  (284.92; 534.74) | 347.87  (296.79; 479.70) | 0.629 | 457.63  (381.98; 573.35) | 328.32  (283.38; 492.30) | 0.080 | 457.63 (292.18; 528.23) | 333.43 (289.74; 480.55) | 0.178 |
| GM-CSF | 0.09  (0.09; 2.06) | 0.09  (0.09; 2.09) | 0.660 | 0.09  (0.09; 1.03) | 0.09  (0.09; 2.09) | 0.496 | 0.09  (0.09; 3.93) | 0.09  (0.09; 1.89) | 0.571 | 0.09  (0.09; 0.09) | 0.09  (0.09; 2.04) | 0.462 |
| IFN- γ | 3.92  (2.58; 8.69) | 2.80  (2.58; 4.16) | 0.103 | 3.64  (2.58; 8.32) | 2.67  (2.58; 5.52) | 0.263 | 6.86  (2.58; 8.36) | 2.67 (2.58; 4.35) | 0.056 | 6.86  (2.58; 13.88) | 2.73  (2.58; 4.53) | 0.155 |
| IP-10 | 1093.28 (688.24; 1852.55) | 896.95 (597.72; 1182.70) | 0.177 | 993.63  (660.10; 1513.53) | 969.08  (638.48; 1401.93) | 0.834 | 995.24  ( 597.72; 1435.43) | 977.58  (667.61; 1513.53) | 0.912 | 1370.52 (696.74; 1513.19) | 972.42 (614.85; 1474.65) | 0.314 |
| MCP-1 | 25.82  (20.01; 36.29) | 21.45  (16.01; 29.66) | 0.224 | 27.83  (21.33; 39.72) | 21.85  (17.04; 29.35) | **0.016** | 28.21  (21.45; 36.29) | 23.60  (17.80; 34.89) | 0.594 | 28.21  (17.55; 40.55) | 23.53  (18.42; 31.99) | 0.268 |
| MIP-1α | 2.89  (1.75; 4.74) | 2.20  (1.61; 2.85) | 0.349 | 2.47  (1.58; 4.83) | 2.60  (1.62; 3.94) | 0.634 | 3.57  (2.68; 9.31) | 2.10  (1.54; 4.08) | **0.013** | 3.57  (2.75; 6.66) | 2.13  (1.54; 4.20) | **0.023** |
| PDGF-BB | 2571.49 (1900.14; 3611.82) | 3178.99 (2211.94; 4290.83) | 0.173 | 2153.35  (1609.41; 3451.73) | 3178.99  (2320.38; 4208.90) | 0.0574 | 2073.61 (1586.55; 3351.54) | 3120.67 (1956.17; 3649.08) | 0.290 | 2073.61 (1586.55; 3351.54) | 3120.67 (1968.40; 3745.52) | 0.178 |
| MIP-1β | 127.55 (109.31; 138.88) | 131.04 (114.72; 144.69) | 0.519 | 127.26  (114.28; 143.54) | 128.26  (115.36; 143.08) | 0.6245 | 125.82  (104.80; 134.46) | 127.84 (114.76; 144.47) | 0.290 | 122.57 (93.90; 134.46) | 128.10 (114.76; 143.66) | 0.348 |
| RANTES | 10960.80 (9811.60; 12495.46) | 11981.98 (10534.02; 14682.49) | 0.089 | 11081.79  (9541.61; 12996.99) | 11425.05  (9987.16; 13910.26) | 0.4418 | 11080.78 (8905.48; 12780.70) | 11334.82 (10075.79; 13872.56) | 0.754 | 10596.68 (8905.48; 11425.05) | 11691.54 (10075.79; 13872.56) | 0.158 |
| TNF- α | 23.21  (20.90; 30.15) | 22.00  (19.46; 31.46) | 0.585 | 23.07  (20.88; 32.80) | 22.00  (19.46; 29.36) | 0.2687 | 29.36  ( 22.44; 37.08) | 22.00  (20.10; 28.97) | **0.020** | 23.90  (20.85; 31.46) | 22.16  (20.11; 29.86) | 0.582 |
| VEGF | 102.64 (73.59; 157.42) | 85.78  (2.40; 173.58) | 0.359 | 103.06  (65.79; 151.79) | 88.60  (32.33; 173.58) | 0.5502 | 107.21  (76.78; 359.25) | 87.19  (45.81; 153.28) | 0.122 | 69.99  (45.81; 146.17) | 96.93  (60.22; 183.81) | 0.318 |

**Table S4.** Cytokine levels according to response to treatment with bortezomib-based regimens. Data are presented as median and IQR.

| **Cytokine**  [pg/mL] | **Treatment response** | | **p** | **Treatment response** | | **p** |
| --- | --- | --- | --- | --- | --- | --- |
|  | **CR**  **N=25** | **Less than CR N=46** |  | **≥VGPR**  **N=41** | **Less than VGPR**  **N=30** |  |
| IL-1 β | 0.87  (0.67; 1.44) | 1.11  (0.79; 1.40) | 0.401 | 0.87  (0.70; 1.23) | 1.21  (0.91; 1.40) | 0.067 |
| IL-1ra | 96.35  (84.33; 196.00) | 95.06  (76.68; 167.15) | 0.917 | 98.94  (84.44; 173.29) | 86.81  (69.69; 190.37) | 0.508 |
| IL-2 | 5.47  (3.81; 8.09) | 5.46  (4.57; 6.59) | 0.869 | 6.13  (4.06; 8.09) | 5.08  (4.53; 5.93) | 0.213 |
| IL-4 | 5.78  (3.70; 8.04) | 5.85  (3.58; 7.42) | 0.602 | 5.78  (3.41; 7.79) | 5.91  (4.61; 7.92) | 0.547 |
| IL-5 | 20.46  (15.69; 29.20) | 19.09  (13.34; 25.85) | 0.641 | 20.89  (14.52; 29.87) | 19.09  (13.56; 24.73) | 0.374 |
| IL-6 | 2.92  (1.61; 4.96) | 3.71  (1.77; 5.50) | 0.581 | 3.11  (1.48; 6.25) | 3.71  (2.07; 5.24) | 0.712 |
| IL-7 | 22.34  (14.06; 23.07) | 22.77  (15.42; 27.82) | 0.590 | 22.34  (14.06; 24.31) | 22.77  (16.33; 28.86) | 0.325 |
| IL-8 | 8.73  (5.61; 12.81) | 9.11  (6.11; 14.26) | 0.544 | 9.45  (5.25; 13.74) | 9.05  (6.41; 14.26) | 0.617 |
| IL-9 | 459.02  (394.42; 497.73) | 479.63  (431.98; 521.17) | 0.207 | 457.36  (397.12; 497.73) | 494.25  (435.17; 550.02) | **0.045** |
| IL-10 | 8.84  (0.70; 11.78) | 0.70  (0.70; 9.65) | 0.102 | 8.03  (0.70; 11.78) | 0.70  (0.70; 9.65) | 0.346 |
| IL-12 (p70) | 0.52  (0.52; 1.26) | 0.52  (0.52; 1.96) | 0.573 | 0.52  (0.52; 1.26) | 1.26  (0.52; 1.61) | 0.308 |
| IL-13 | 2.16  (1.25; 3.25) | 1.99  (1.24; 2.70) | 0.611 | 2.09  (1.24; 2.81) | 1.99  (1.55; 2.70) | 0.919 |
| IL-15 | 38.46  (0.73; 57.26) | 38.43  (0.73; 63.14) | 0.898 | 38.46  (0.73; 61.65) | 26.94  (0.73; 60.44) | 0.617 |
| IL-17 | 22.27  (14.22; 28.13) | 18.88  (15.43; 23.44) | 0.451 | 18.84  (14.22; 25.82) | 20.27  (16.60; 23.39) | 0.924 |
| Eotaxin | 80.85  (53.62; 110.45) | 80.72  (49.35; 100.63) | 0.769 | 80.36  (49.06; 103.43) | 85.14  (64.06; 122.48) | 0.252 |
| FGF basic | 37.70  (33.85; 41.12) | 36.76  (31.57; 41.72) | 0.677 | 37.70  (30.82; 41.78) | 36.76  (32.11; 40.98) | 0.849 |
| G-CSF | 386.56  (294.30; 565.95) | 364.43  (297.12; 479.70) | 0.508 | 386.56  (292.18; 565.95) | 364.43  (298.48; 457.63) | 0.474 |
| GM-CSF | 0.09  (0.09; 2.01) | 0.09  (0.09; 2.20) | 0.845 | 0.09  (0.09; 2.31) | 0.09  (0.09; 1.51) | 0.482 |
| IFN- γ | 3.41  (2.55; 8.03) | 3.61  (2.58; 7.10) | 0.336 | 3.42  (2.58; 8.03) | 2.67  (2.58; 6.86) | 0.972 |
| IP-10 | 992.03  (638.48; 1435.43) | 975.76  (696.74; 1382.12) | 0.713 | 975.76  (631.97; 1513.19) | 995.24  (799.42; 1227.27) | 0.962 |
| MCP-1 | 26.64  (17.55; 36.64) | 23.63  (19.09; 30.83) | 1.000 | 23.63  (17.55; 36.64) | 24.43  (19.72; 30.83) | 0.617 |
| MIP-1 α | 3.25  (2.20; 5.26) | 2.07  (1.58; 3.94) | **0.037** | 2.88  (1.86; 5.26) | 2.07  (1.51; 2.89) | **0.019** |
| PDGF-BB | 3178.99  (2094.45; 3611.82) | 2655.84  (1651.84; 3645.86) | 0.590 | 3223.46  (2094.45; 3652.29) | 2336.52  (1651.84; 3454.11) | 0.206 |
| MIP-1 β | 123.64  (109.02; 135.94) | 131.04  (116.97; 144.69) | 0.101 | 125.82  (110.30; 135.94) | 135.36  (126.36; 147.31) | **0.022** |
| RANTES | 10661.32  (9319.77; 11981.98) | 11858.24  (10132.57; 13915.11) | 0.141 | 10823.59  (9915.60; 12449.44) | 11972.89  (10132.57; 14243.54) | 0.113 |
| TNF- α | 23.90  (20.85; 33.95) | 22.44  (20.13; 28.94) | 0.214 | 23.90  (20.13; 32.42) | 22.00  (20.52; 28.59) | 0.328 |
| VEGF | 95.24  (57.45; 149.14) | 95.37  (62.99; 157.42) | 0.917 | 101.29  (61.59; 173.58) | 85.78  (45.81; 136.02) | 0.311 |
